# Dual-Emission Carbon-Dot Ratiometric Fluorescence Sensor for Morphine Recognition in Biological Samples

**DOI:** 10.3390/bios13010143

**Published:** 2023-01-15

**Authors:** Qinhong Yin, Yijie Wang, Xuerong Li, Dezhi Yang, Yaling Yang, Cheng Yang, Yanqin Zhu

**Affiliations:** 1 Faculty of Narcotics Control, Yunnan Police College, Kunming 650223, China; 2 Faculty of Life Science and Technology, Kunming University of Science and Technology, Kunming 650500, China; 3 Research Center for Analysis and Measurement, Kunming University of Science and Technology, Kunming 650093, China

**Keywords:** carbon dots, fluorescent sensor, dual-emission, morphine

## Abstract

Herein, a novel nitr[ogen-doped carbon dot (N-CD) fluorescence sensor with a dual emission ratio is developed using the microwave-assisted synthesis of m-phenylenediamine and spermidine. As a result of the fluorescence inner filtration effect (IFE) effect between morphine (MOR) and N-CD, the blue fluorescence of N-CDs at 350 nm was reduced in the presence of MOR, whereas the fluorescence of N-CDs at 456 nm increased substantially. The results demonstrated that the approach has a tremendous potential and that the linear range of MOR detection is 0.25–25 µg/mL, with a 71.8 ng/mL detection limit. Under UV light, the blue fluorescent system is easily visible to the naked eye. More significantly, the sensor proved successful in providing satisfactory results for the speciation measurement of MOR in a variety of biological samples.

## 1. Introduction

An essential opium alkaloid known as morphine (MOR) is a natural source of a phenanthrene derivative purified from the *poppy* plant [1]. Clinically, it helps patients with pain relief, particularly chronic cancer symptoms. Regrettably, MOR can, like all opioids, lead to addiction and even death as a result of respiratory failure [2,3,4,5]. Overdose deaths caused by opioids claimed over 100,000 lives in the United States for the 12 months ending in April 2021, a 28.5% increase from the year before [6]. To monitor MOR levels in biological samples and prevent overdose or abuse-induced toxicity, morphine analysis is required in the healthcare and forensic domains [7].

It is generally accepted that MOR diagnosis uses two strategies: initial on-site screening and laboratory validation. The majority of the initial screening process involves qualitative analysis, which can immediately determine whether specimens contain morphine. The commonly used screening techniques include immunoassay, spectroscopy, and molecular imprinting [8,9,10]. These initial screening methods, although simple and fast, cannot be quantitatively analyzed and are also prone to false positive or false negative phenomena. For laboratory confirmation, samples are sent to the laboratory for further quantitative analysis using large instruments such as HPLC-MS (High-Performance Liquid Chromatography-Mass Spectrometry) [11,12] and GC-MS (Gas Chromatography-Mass Spectrometry) [13,14]. However, these instruments are bulky and require professional personnel to operate, so they cannot be used for rapid detection in the field of front-line law enforcement.

The fluorescence analysis method has been used more and more in field rapid detection due to its features of convenient operation, simple equipment, high sensitivity, and strong selectivity [15]. Carbon dots (CDs), generally termed semiconductor nanocrystals, are zero-dimensional nanocrystals, and the size is no more than twice the exciton Bohr radius of the corresponding semiconductor material [16]. When the size reaches a critical value, the energy will be completely quantized, showing quantum characteristics, so it is called quantum dots. As a novel form of fluorescent semiconductor, quantum dots have many unique properties compared with traditional fluorescent dyes, such as good photostability, adjustable fluorescence emission wavelength, high fluorescence intensity, and good biocompatibility [17]. Because of their unique optical properties, they have gradually replaced the traditional organic fluorescent dyes and are a relatively ideal fluorescent nanomaterial. CDs have been used in detection and analysis more frequently due to their excellent fluorescence performance [18,19,20].

As the name implies, CDs are used in chemical sensors because of their molecular recognition function. However, fluorescent chemical sensors are usually interfered with or affected by sensor concentration, stability of detector or light source, and coexistence of components in a complex sample matrix [21]. The double emission ratio fluorescence method can avoid the above problems by using ratio fluorescence to detect the target [22]. It is still relatively uncommon to use carbon dots in the development of ratio fluorescence probes and to use CDs further in the detection of illegal drugs in biological samples.

In this study, novel carbon dots were synthesized from m-phenylenediamine and spermidine as the source of N atoms by microwave. Due to the improved inhibition efficiency attributed to the fluorophore’s inner filter effect of the nanoparticles in the occurrence of MOR, the fluorescence detector was modified for the analysis of MOR. For the highly selective MOR measurement in biological samples, the dual emission ratio fluorescence sensor has been successfully employed.

## 2. Materials and Methods

### 2.1. Chemicals

All reagents and chemicals were analytical grade (⩾99.0% purity) and were utilized as received without additional purification. The Ministry of Public Security’s Key Laboratory of Narcotics Assay and Control Technology supplied MOR, heroin and methylamphetamine standards (⩾99.5% purity). Spermidine (99.0% purity) and m-phenylenediamine (99.0% purity) were provided by Yuanye Biotechnology Co., Ltd., and Aladdin Biochemical Technology Co., Ltd., both of Shanghai, China. Siens Biochemical Technology Co., Ltd. and Maclean Biochemical Technology Co., Ltd. (Shanghai, China) provided the amino acids (⩾98.0% purity) used in the interference test (Tianjin, China). All other chemicals were purchased from Zhiyuan Chemical Reagent Co., Ltd. and Fengchuan Chemical Reagent Technologies (Tianjin, China). Using the Milli-Q filtration apparatus (Millipore, Bedford, MA, USA), double-distilled water (18.2 MΩ cm) was utilized as the input to generate deionized water.

### 2.2. Instruments

A quartz cell (1 cm × 1 cm)-equipped Agilent G9800A Cary Eclipse fluorescence spectrophotometer (USA) was applied to record the fluorescence spectrum. The emission and excitation monochromatic slit widths were both fixed at 10 nm. A TENSOR-27 FTIR spectrometer was used to record the FT-IR spectrum (Bruker, Bremen, Germany). Utilizing a UV-2550 spectrophotometer, the ultraviolet-visible (UV-vis) spectrum was recorded (Shimadzu, Kyoto, Japan). PHI5000 Versa Probeqy-II with monochromatized Al K light was used to characterize X-ray photoelectron spectroscopy (XPS) (ULVAC-PHI, Kanagawa, Japan). X-ray diffraction (XRD) patterns were recorded by a PANalytical X’pert^3^ powder diffractometer using Ni-filtered Cu K*a* radiation. Using a transmission electron microscope, TecnaiG2 F30 S-Twin (FEI, Hillsboro, OR, USA) was applied to analyze the size and appearance of carbon dots. Carbon dots were synthesized using the Analytik-Jena TOPwave microwave-assisted digesting system (Jena, Germany). Using a Leici PHS-3 digital pH meter, the pH was controlled (Shanghai, China).

### 2.3. Preparation of Real Samples and Standard Solutions

In this study, blood samples were collected from healthy individuals. To collect serum at 4 °C for the plasma preparation, blood samples were centrifuged at 4000 rpm for 30 min. In water, a stock standard solution of MOR (100 μg/mL) was prepared. The stock standard solutions were diluted with deionized water to formulate working solutions. All solutions were kept in a freezer at 4 °C.

### 2.4. Preparation of N-CDs

**Figure 1:**
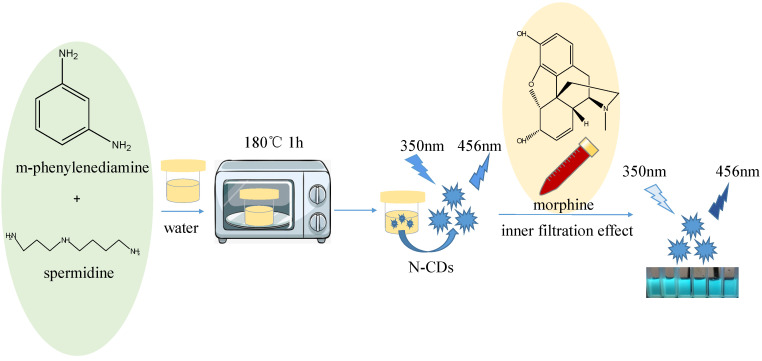
A schematic illustrating the synthesis and morphine detection of N-CDs.

The fluorescent N-CD schematic diagram for the synthesis process is shown in Figure 1. Spermidine (0.4 g) and m-phenylenediamine (0.4 g) were precisely weighed, dissolved in 40 mL of deionized water, and then heated at 180 °C for one hour in a microwave. Using a 0.22 μm filter membrane, the sample was filtered, and the filtrate was collected for dialyzing. The fractions corresponding to 3 kDa were ultrafiltered from N-CDs and studied in our work.

### 2.5. Detection of MOR with the Ratiometric Sensor

At room temperature, 100 µL of N-CDs solution and 20 µL of serum with various concentrations of MOR were carefully mixed. Under the optimized conditions, the pH was adjusted to 8 with citrate-disodium hydrogen phosphate buffer. Next, the mixture was diluted to 4 mL with deionized water and heated in a water bath at 44 °C to maximize the sensitivity. The final MOR concentrations were 0.25, 0.5, 1.0, 1.25, 2.5, 5, 10 and 25 µg/mL. Then, with excitation at 310 nm, fluorescence spectra in the wavelength range of 330–470 nm were obtained. This sensing system’s MOR selectivity was evaluated using NO^2−^, HCO_3_^−^, K^+^, Na^+^, Mg^2+^, Cu^2+^, K^+^, Fe^2+^, Mn^2+^, Zn^2+^ and other amino acids, such as Tyr, Gly, Try, Leu, Met, Glu, Lys, and Cys.

## 3. Results and Discussion

### 3.1. Characterization Results of N-CDs

**Figure 2:**
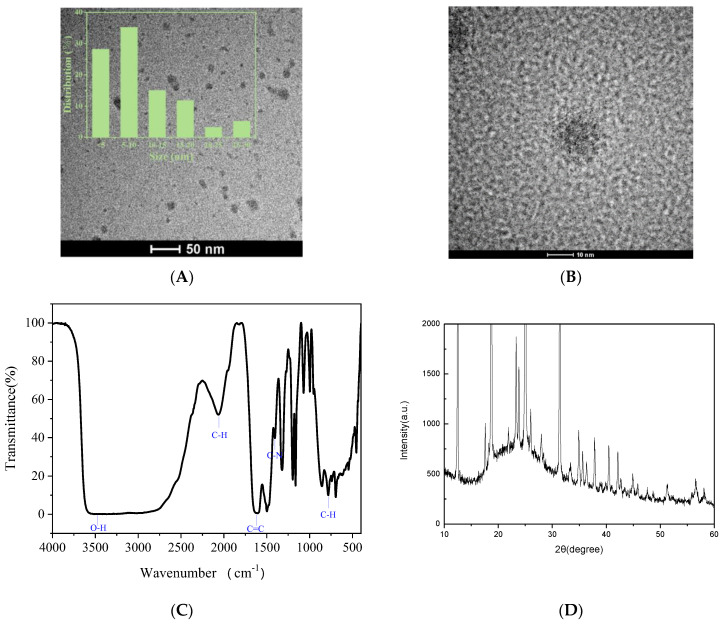
The analysis of N-CDs using (**A**) particle size distribution and (**B**) TEM images at different scales; (**C**) FT-IR; (**D**) XRD.

TEM was used to analyze the nanostructure of N-CDs. N-CD TEM images at various scales are displayed in Figure 2A,B. As shown in Figure 2A, the transmission TEM images show good monodispersity and good size homogeneity of N-CDs, clearly indicating that these nanoparticles are almost spherical with an average size of 5 nm. However, neither Figure 2A nor Figure 2B showed any obvious lattice fringes, suggesting that the crystallinity of the carbon dots is weak, which is in accordance with previous research [23,24].

The N-CD FT-IR spectra are shown in Figure 2C. The stretching vibrations of O-H and N-H are associated with the low-intensity band at 3472 cm^−1^. The C-H stretching and bending vibrations were demonstrated by the peaks at 2065 cm^−1^ and 732 cm^−1^, respectively [25]. The C=C and C-N bending vibrations are correlated to the peaks at 1605 and 1424 cm^−1^, respectively [26]. These results confirmed the existence of the -NH2, C-N, and C=C groups, which enhanced the solubility of N-CDs in water.

Figure 2D shows the XRD patterns of the obtained material. As can be seen, it has an amorphous structure with a broad peak at 2θ = 21.22° that is clearly related to the amorphous nature of C-dots.

**Figure 3:**
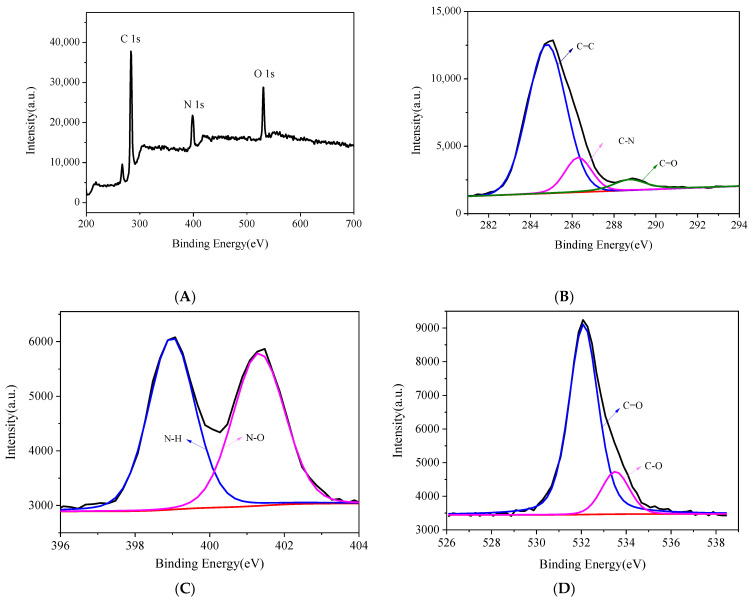
(**A**) XPS spectra of N-CDs, high resolution (**B**) C 1s, (**C**) N 1s, and (**D**) O 1s peaks of N-CDs.

XPS was applied to investigate the chemical groups on the surface of N-CDs. C1s, N1s, and O1s concentration levels are the origin of the peaks in the spectrum at 283.2, 399.2, and 530.4 eV (Figure 3A). Three peaks in the C1s spectra can be assigned to the C=C, C-N, and C=O groups with energies of 284.8, 286.2, and 288.8 eV, respectively (Figure 3B) [27]. Two peaks in the N1s spectra were observed at 399.2 and 401.3 eV (Figure 3C), and they might belong to N-H and N-O bond types. Two major peaks from the C=O and C-O groups can be observed at 532.0 and 533.7 eV, according to further analysis of the O1s spectra (Figure 3D) [28]. Additionally, quantum yield (QY) of N-CDs was determined based on the relative method by using quinine sulfate as reference (dissolved in 0.1 M H_2_SO_4_, QY = 54.6%) [29]. According to the reference calculation formula, the N-CD quantum yield was 12.95%.

### 3.2. Optical Properties of N-CDs

**Figure 4:**
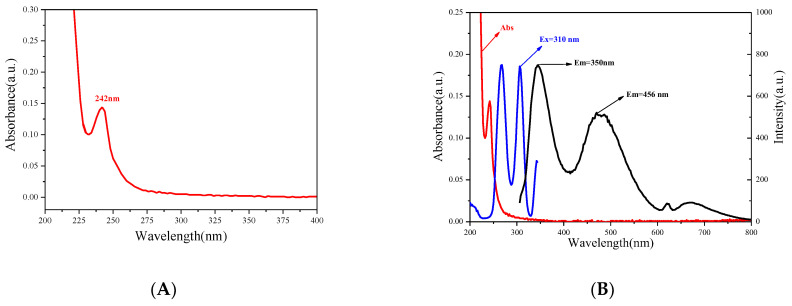
(**A**) UV-Vis absorption spectra of N-CDs; (**B**) fluorescence spectra of N-CDs.

Figure 4A shows the N-CDs’ UV-vis spectrum. The π-π* transitions are responsible for the shoulder peak at 242 nm [30]. Besides the peak at 242 nm, no sharp absorption peaks were present for N-CDs, except for the display of a long absorption edge, which was extended from 275 to 400 nm. Using the same 310 nm excitation wavelength, N-CDs in this sensor system displayed two emission peaks (350 nm and 456 nm) (Figure 4B).

### 3.3. Effect of Solution pH

**Figure 5:**
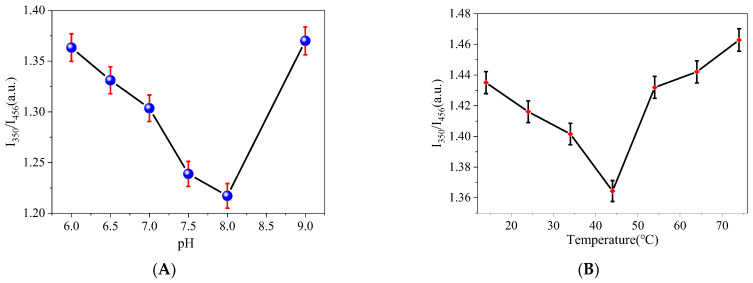
Effect of (**A**) pH under the same conditions (system temperature: 44 °C; N-CDs: 100 µL; MOR concentration: 10 μg/mL; Ex: 310 nm; Em: 350 nm and 456 nm) and (**B**) system temperature under the same conditions (pH: 8.0; N-CDs: 100 µL; MOR concentration: 10 μg/mL; Ex: 310 nm; Em: 350 nm and 456 nm).

Fluorophore ionization has a significant impact on how much light is emitted. Since the I_350_/I_456_ value decreases with increasing concentration of MOR, it is necessary to adjust the solution pH to maximize a method’s sensitivity. To maximize a method’s sensitivity, it is necessary to adjust the pH of a solution. The pH ranged in this study from 6.0 to 9.0. I_350_/I_456_’s fluorescence ratio increased from pH 8.0 to 9.0 after declining from pH 6.0 to 8.0. (Figure 5A). Therefore, before testing, the pH value of the test solution must be adjusted to 8.0.

### 3.4. System Temperature

Temperature has an effect on the return of excited electrons to the ground state to produce changes in fluorescence intensity. Thus, the effects of system temperatures of 14, 24, 34, 44, 54, 64, and 74 °C on the fluorescence intensity were explored (Figure 5B). The results show that the fluorescence ratio of I_350_/I_456_ decreased from 14 °C to 44 °C and then increased when the temperature increased from 44 °C to 74 °C. Considering this comprehensively, the temperature of the system was chosen at room 44 °C. The temperature change feature could be attributed to the temperature-enhanced population of non-radiative channels of surface (trap/defect) states. More non-radiative channels would be activated at a higher temperature, and more excited electrons returned to the ground state via a non-radiative process, resulting in decreased fluorescence intensity [31,32].

### 3.5. Effect of Interfering Ions and Substances

**Figure 6:**
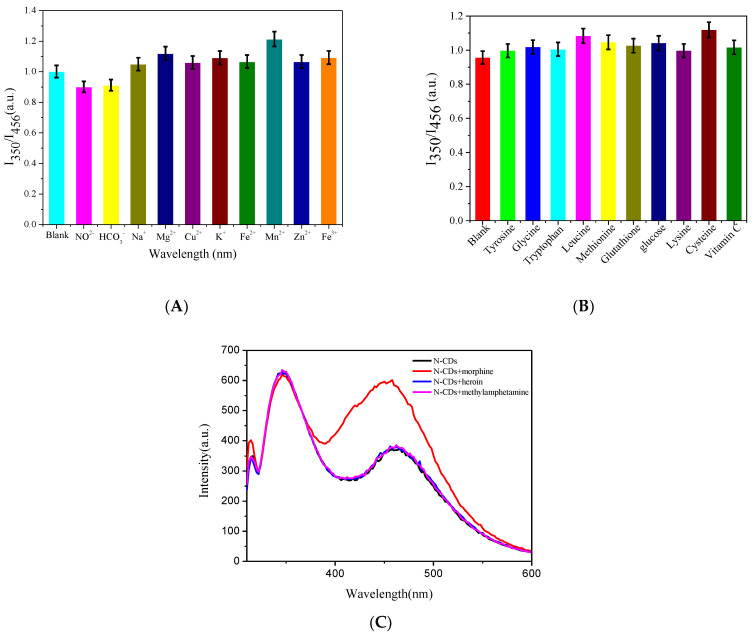
Effect of (**A**) interfering ions and (**B**) interfering substances under the same conditions (pH: 8.0; system temperature: 44 °C; N-CDs: 100 µL; MOR concentration: 10 μg/mL; Ex: 310 nm; Em: 350 nm and 456 nm) (n = 3); (**C**) fluorescence spectra of MOR and its analogues (pH: 8.0; system temperature: 44 °C; N-CDs: 100 µL; concentration of MOR and its analogues: 10 μg/mL).

An innovative fluorescence probe must have excellent selectivity. The effects of different ions (NO^2−^, HCO_3_^−^, Na^+^, Mg^2+^, Cu^2+^, K^+^, Fe^2+^, Mn^2+^, Zn^2+^, Fe^3+^) and interfering substances (tyrosine, glycine, tryptophan, leucine, methionine, glutathione, glucose, lysine, cysteine and vitamin C) added in the same proportion (100 μg/mL) on the fluorescence signal of the morphine+N-CDs system were explored. Experiments were carried out three times in regard to the results displayed in Figure 6A,B. These interfering ions and interfering substances did not affect the system. The systematic execution of N-CDs concerning their selectivity towards MOR was also conducted against some of the very common interfering MOR analogues such as heroin and methylamphetamine. It was found that morphine significantly enhanced the fluorescence of N-CDs at 466 nm, while heroin and methamphetamine had no effect on N-CDs at 466 nm (Figure 6C). The above results confirm the good selectivity of the ratiometric fluorescence sensor toward MOR.

### 3.6. Method Validation

**Figure 7:**
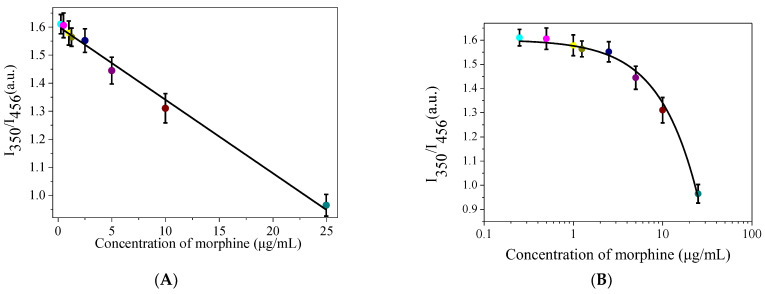
(**A**) A linear plot of I_350_/I_456_ versus the concentration of morphine in the range of 0.25–25 μg/mL (n = 3); (**B**) Fitted curves of I_350_/I_456_ versus the morphine concentration plotted on a log scale.

The concentration of MOR displayed a consistent pattern as a function of I_350_/I_456_ in the range of 0.25–25 μg/mL (I_350_/I_456_ = 1.6027 − 0.02622C, R^2^ = 0.9910) under optimal conditions (Figure 7). Error bars in the calibration curve were obtained from three parallel measurements. The method’s LOD was determined to be 71.8 ng/mL using the formula 3 s/K (s is the continuous determination standard deviation of 10 blanks, and K is the slope of the calibration trendline), and the relative standard deviation (RSD) was 4.6% (c = 5 μg/mL, n = 8), in accordance with the IUPAC standard [33]. Based on this, the limit of quantitation (LOQ) of MOR determined by this sensor was calculated to be 0.239 μg/mL by 10 s/k. This approach exhibited a comparable detection limit to earlier reported MOR probes based on CDs, but it had a higher selectivity [34,35,36,37,38].

### 3.7. Analysis of Real Samples

Considering the complexity of blood samples, we added morphine to blood samples in this study to verify the accuracy and anti-interference of the method. Actual blood samples from patients were tested using the standardized calibration curve. The suggested method was used to determine the amounts of MOR in various blood samples (Table 1). The innovative fluorescent approach used had a recovery of between 93.8% and 103.3% with RSDs of under 6%. The analytical data of several strategies for MOR detection are evaluated in Table 2. The outcomes reveal that the proposed approach can be used to swiftly test blood for illegal drugs using the methods outlined. The results confirmed that the proposed method showed good anti-interference and accuracy.

### 3.8. Sensing Mechanism of Ratiometric Nanosensor towards MOR

**Figure 8:**
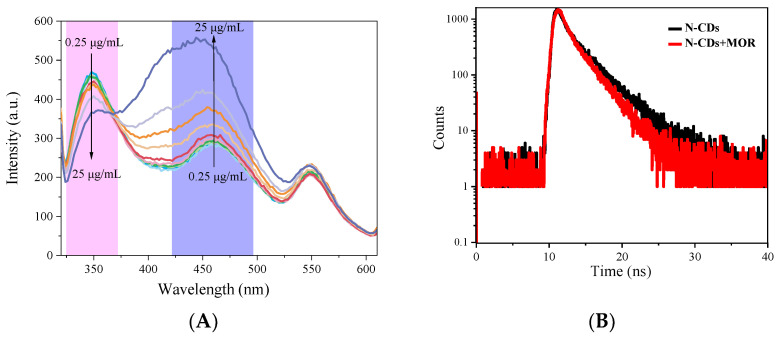

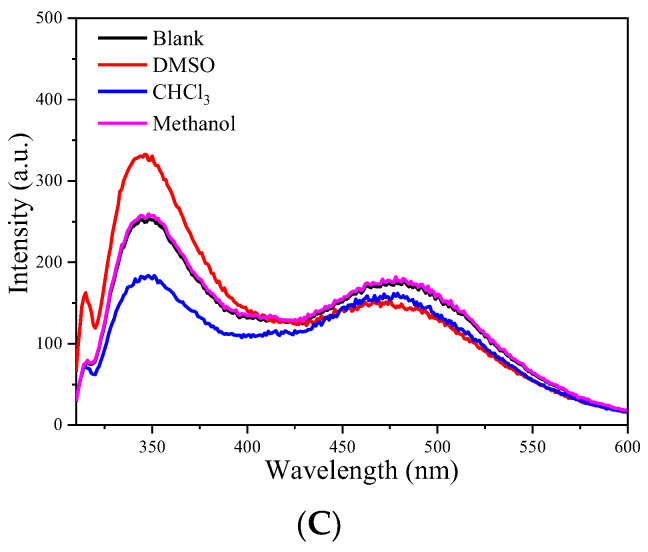
(**A**) Fluorescence spectra of N-CD solution with different concentrations of morphine. (**B**) The fluorescence lifetime curve of N-CDs and N-CDs + morphine. (**C**) The fluorescence spectra of N-CDs in different apolar solvents.

We performed a series of tests to examine the effects of various MOR substances in the N-CDs system, and the results are shown in Figure 8A. These tests were carried out to investigate the potential mechanism of the N-CDs/MOR based fluorescence sensing technique for MOR analysis. The fluorescence of N-CDs at 350 nm was reduced when the MOR was introduced, whereas the fluorescence of N-CDs at 456 nm was substantially enhanced. The absorbance peak of MOR aligns with the emission spectrum of N-CDs, as shown in Figure 8A, demonstrating the possible existence of an inner filtration effect (IFE) or Förster resonance energy transfer (FRET) between N-CDs and MOR [39,40]. As a result, the intensity of the N-CDs fluorescence emission gradually decreased with the addition of morphine. As a result of the comparatively small size distribution of the resulting N-CDs, Figure 4B also exhibits a narrow band at 300–400 nm in the emission spectra. To further confirm the sensing mechanism, a fluorescence lifetime experiment was conducted [41]. Figure 8B shows the fluorescence lifetime graph. The average lifetime of fluorescence is 2.25 ns (χ2 = 0.90), while the lifetime components of N-CDs are τ1 = 0.83 ns (26.15%) and τ2 = 2.42 ns (73.85%). The mean fluorescence lifetime is 2.83 ns (χ2 = 0.94), and the lifetime components after the addition of morphine are τ1 = 0.86 ns (23.76%) and τ2 = 3.01 ns (76.24%). These results show that the fluorescence lifetime is essentially unaffected by the presence or absence of MOR. This indicates that IFE, not FRET, causes fluorescence quenching because the donor’s PL lifetime is constant during IFE but seems to change significantly during FRET. As a result, we can conclude that MOR and N-CDs have an IFE effect [42].

Since FRET is not affected by the polarity of the solvent, changing the polarity of the solution has little effect on the quenching efficiency based on FRET. Therefore, we also studied the interaction of N-CDs in different apolar solvents (Figure 8C). The experimental results show that the fluorescence is dramatically changed in dimethylsulfoxide (DMSO) and chloroform (CHCl_3_). This further confirms that the interaction mechanism between N-CDs and MOR might be due to IFE.

## 4. Conclusions

In this study, a simple and accurate ratiometric fluorescence sensor for MOR measurement between N-CDs and MOR was developed. Based on the FRET effect, the addition of MOR further inhibited the fluorescence of N-CDs. High sensitivity, superior selectivity, rapid detection, and an expanded linear response range were all promising characteristics of the dual-emission carbon-dot ratiometric fluorescence sensing device. The detection of MOR in actual blood samples was evidence of the developed method.

## Figures and Tables

**Table 1:** Quantification of MOR in samples (n = 3).

Samples	Added (μg/mL)	Found (μg/mL)	Recovery (%)	RSD (%)
Blood (female)	-	N. D. ^a^	-	-
	2.495	2.411	96.7	5.6
	4.990	5.153	103.3	4.3
Blood (male)	-	N. D. ^a^	-	-
	2.495	2.515	100.8	3.5
	4.990	4.683	93.8	4.9

^a^ Not detected.

**Table 2:** The overview of analytical data of the reported methods for the analysis of MOR.

Materials	Detection Method	Linearity Range	LOD	Reference
graphene quantum dots	voltammetric electrode	0–3.5 μM	0.06 μM	[34]
chiral colloidal CdSe quantum dots	fluorescence enhancement	/	0.06 μM	[35]
graphene quantum dots	fluorescence enhancement	0–33 μM	0.5 μg/mL	[36]
anti-morphine antibody-labeled C-Dots	fluorescence immunoassay	3.2 × 10^−4^–10 mg/L	3.0 × 10^−4^ mg/L	[37]
N,Cl-CDs	fluorescence enhancement	0.15–280.25 μg/mL	46.5 ng/mL	[38]
N-CDs	fluorescence quenching	0.25–25 μg/mL	71.8 ng/mL	This work

## Data Availability

Not applicable.
